# Boundaries mediate long-distance interactions between enhancers and promoters in the *Drosophila Bithorax* complex

**DOI:** 10.1371/journal.pgen.1007702

**Published:** 2018-12-12

**Authors:** Nikolay Postika, Mario Metzler, Markus Affolter, Martin Müller, Paul Schedl, Pavel Georgiev, Olga Kyrchanova

**Affiliations:** 1 Department of the Control of Genetic Processes, Institute of Gene Biology Russian Academy of Sciences, Moscow, Russia; 2 Biozentrum, University of Basel, Basel, Switzerland; 3 Department of Gene Expression Regulation in Development, Institute of Gene Biology Russian Academy of Sciences, Moscow, Russia; 4 Department of Molecular Biology, Princeton University, Princeton, New Jersey, United States of America; University of Lausanne, SWITZERLAND

## Abstract

*Drosophila* bithorax complex (BX-C) is one of the best model systems for studying the role of boundaries (insulators) in gene regulation. Expression of three homeotic genes, *Ubx*, *abd-A*, and *Abd-B*, is orchestrated by nine parasegment-specific regulatory domains. These domains are flanked by boundary elements, which function to block crosstalk between adjacent domains, ensuring that they can act autonomously. Paradoxically, seven of the BX-C regulatory domains are separated from their gene target by at least one boundary, and must “jump over” the intervening boundaries. To understand the jumping mechanism, the *Mcp* boundary was replaced with *Fab-7* and *Fab-8*. *Mcp* is located between the *iab-4* and *iab-5* domains, and defines the border between the set of regulatory domains controlling *abd-A* and *Abd-B*. When *Mcp* is replaced by *Fab-7* or *Fab-8*, they direct the *iab-4* domain (which regulates *abd-A*) to inappropriately activate *Abd-B* in abdominal segment A4. For the *Fab-8* replacement, ectopic induction was only observed when it was inserted in the same orientation as the endogenous *Fab-8* boundary. A similar orientation dependence for bypass activity was observed when *Fab-7* was replaced by *Fab-8*. Thus, boundaries perform two opposite functions in the context of BX-C–they block crosstalk between neighboring regulatory domains, but at the same time actively facilitate long distance communication between the regulatory domains and their respective target genes.

## Author summary

*Drosophila* bithorax complex (BX-C) is one of a few examples demonstrating *in vivo* role of boundary/insulator elements in organization of independent chromatin domains. BX-C contains three *HOX* genes, whose parasegment-specific pattern is controlled by *cis*-regulatory domains flanked by boundary/insulator elements. Since the boundaries ensure autonomy of adjacent domains, the presence of these elements poses a paradox: how do the domains bypass the intervening boundaries and contact their proper regulatory targets? According to the textbook model, BX-C regulatory domains are able to bypass boundaries because they harbor special promoter targeting sequences. However, contrary to this model, we show here that the boundaries themselves play an active role in directing regulatory domains to their appropriate *HOX* gene promoter.

## Introduction

The three homeotic (HOX) genes in the *Drosophila* Bithorax complex (BX-C), *Ultrabithorax* (*Ubx*), *abdominal-A* (*abd-A*) and *Abdominal-B* (*Abd-B*), are responsible for specifying cell identity in parasegments (PS) 5–14, which form the posterior half of the thorax and all of the abdominal segments of the adult fly [1–3]. Parasegment identity is determined by the precise expression pattern of the relevant HOX gene and this depends upon a large *cis*-regulatory region that spans 300 kb and is subdivided into nine PS domains that are aligned in the same order as the body segments in which they operate [4–6]. *Ubx* expression in PS5 and PS6 is directed by *abx/bx* and *bxd/pbx*, while *abd-A* expression in PS7, PS8, and PS9 is controlled by *iab-2*, *iab-3*, and *iab-4* [7–10]. *Abd-B* is regulated by four domains, *iab-5*, *iab-6*, *iab-7* and *iab-8*, which control expression in PS10, PS11, PS12 and PS13 respectively [6,11,12].

**Fig 1:**
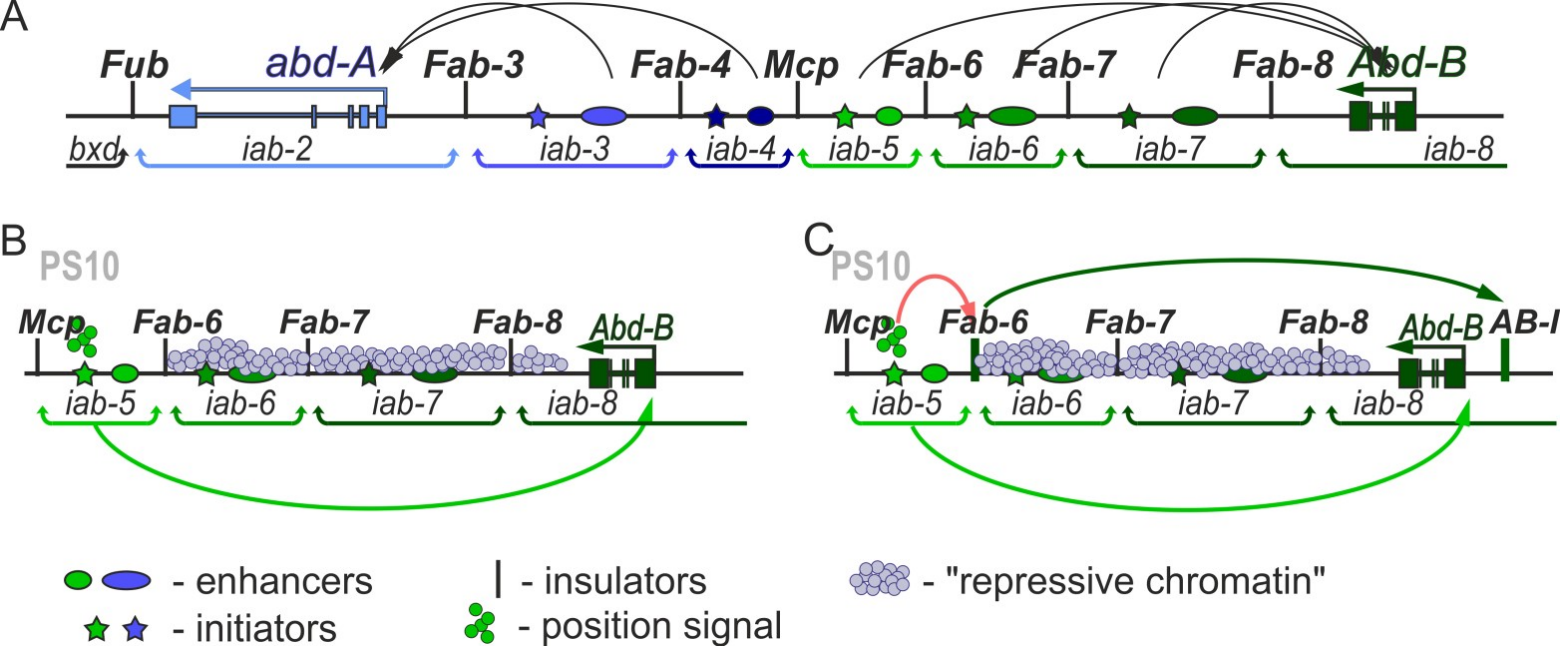
Models of an enhancer–promoter interactions in *BX-C*. (A) Scheme of the regulatory region of the distal part of the BX-C. Horizontal arrows represent transcripts for *abd-A* (blue) and *Abd-B* (green). *iab* enhancers are shown as ovals color-coded with respect to the gene they control (darker shades of color indicate higher expression levels). The black arc arrows are a graphical illustration of the targeting of each *cis-*regulatory domain to the *abd-A* or *Abd-Bm* promoter. Vertical lines mark boundaries (*Fub*, *Fab-3*, *Fab-4*, *Mcp*, *Fab-6*, *Fab-7*, and *Fab-8*) of regulatory *iab* domains which are delimited by brackets behind the map. (B) and (C) Schematic representation of the models explaining interaction of the *iab* enhancers with the *Abd-B* promoter. (B) After stimulation of the initiator (colored star) by a positional signal (small circles with the same color as initiator), the *iab* enhancer directly interacts with the *Abd-B* promoter. This process is shown as light green arc arrow. Domains without positional signal are repressed (shown as light blue small circles across the domain, indicating "repressive chromatin"). (C) Boundary is directly involved in organization of the enhancer-promoter interactions. There is also a boundary-like element *AB-I* upstream of the *Abd-B* promoter that has communicator activity in bypass assays. Proteins of the positional signal bind with initiator of iab domain, and its active status modifies the boundary activity. This process is shown as red arc arrow. Boundary becomes able to bind proteins responsible for communication with *AB-I*. Interaction is shown as dark green arc arrow. As a result, the iab enhancer is localized in close proximity to the *Abd-B* promoter (shown as light green arc arrow).

Each regulatory domain contains an initiator element, a set of tissue-specific enhancers and Polycomb Response Elements (PREs) and is flanked by boundary/insulator elements (Fig 1A;[13]. *BX-C* regulation is divided into two phases, initiation and maintenance [14,15]. During the initiation phase, a combination of gap and pair-rule proteins interact with initiation elements in each regulatory domain, setting the domain in the *on* or *off* state. In PS10, for example, the *iab-5* domain, which regulates *Abd-B*, is activated by its initiator element, while the more distal *Abd-B* domains, *iab-6* to *iab-8* are set in the *off* state (Fig 1B). In PS11, the *iab-6* initiator activates the domain, while the adjacent *iab-7* and *iab-8* domains are set in the *off* state. Once the gap and pair-rule gene proteins disappear during gastrulation, the *on* and *off* states of the regulatory domains are maintained by Trithorax (Trx) and Polycomb (PcG) group proteins, respectively [16,17].

In order to select and then maintain their activity states independent of outside influence, adjacent regulatory domains are separated from each other by boundary elements or insulators [18–24]. Mutations that impair boundary function permit crosstalk between positive and negative regulatory elements in adjacent domains and this leads to the misspecification of parasegment identity. This has been observed for deletions that remove five of the BX-C boundaries (*Front-ultraabdominal* (*Fub*), *Miscadestral pigmentation* (*Mcp*), *Frontadominal-6* (*Fab-6*), *Frontadominal-7* (*Fab-7*), and *Frontadominal-8* (*Fab-8*)) [6,17,19,20,22,23,25,26].

While these findings indicate that boundaries are needed to ensure the functional autonomy of the regulatory domains, their presence also poses a paradox [27,28]. Seven of the nine BX-C regulatory domains are separated from their target HOX gene by at least one intervening boundary element. For example, the *iab-6* regulatory domain must “jump over” or “bypass” *Fab-7* and *Fab-8* in order to interact with the *Abd-B* promoter (Fig 1A). That the blocking function of boundaries could pose a significant problem has been demonstrated by experiments in which *Fab-7* is replaced by heterologous elements such as *scs*, *gypsy* or multimerized binding sites for the architectural proteins dCTCF, Pita or Su(Hw) [25,29–31]. In these replacements, the heterologous boundary blocked crosstalk between *iab-6* and *iab-7* just like the endogenous boundary, *Fab-7*. However, the boundaries were not permissive for bypass, preventing *iab-6* from regulating *Abd-B*.

A number of models have been proposed to account for this paradox. One is that BX-C boundaries must have unique properties that distinguish them from generic fly boundaries. Since they function to block crosstalk between enhancers and silencers in adjacent domains, an appealing idea is that they would be permissive for enhancer/silencer interactions with promoters (Fig 1B). However, several findings argue against this notion. For one, BX-C boundaries resemble those elsewhere in the genome in that they contain binding sites for architectural proteins such as Pita, dCTCF, and Su(Hw) [23,31–35]. Consistent with their utilization of these generic architectural proteins, when placed between enhancers (or silencers) and a reporter gene, BX-C boundaries block regulatory interactions just like boundaries from elsewhere in the genome [19,36–42]. Similarly, there is no indication in these transgene assays that the blocking activity of BX-C boundaries are subject to parasegmental regulation. Also arguing against the idea that BX-C boundaries have unique properties, the *Mcp* boundary, which is located between *iab-4* and *iab-5*, is unable to replace *Fab-7* [31]. Like the heterologous boundaries, it blocks crosstalk, but it is not permissive for bypass. A second model is that there are special sequences, called promoter targeting sequence (PTS), located in each regulatory domain that actively mediate bypass [43–45]. While the PTS sequences that have been identified in *iab-6* and *iab-7* enable enhancers to “jump over” an intervening boundary in transgene assays, they do not have a required function in the context of BX-C and are completely dispensable for *Abd-B* regulation [18,30].

A third model (Fig 1C) is suggested by transgene “insulator bypass” assays [46,47]. In one version of this assay, two boundaries instead of one are placed in between an enhancer and the reporter. When the two boundaries pair with each other, the enhancer is brought in close proximity to the reporter, thereby activating rather than blocking expression. Consistent with a possible role in BX-C bypass, these pairing interactions can occur over large distances and even skip over many intervening boundaries [48–51]. The transgene assays point to two important features of boundary pairing interactions that are likely to be relevant in BX-C. First, pairing interactions are specific. For this reason boundaries must be properly matched with their neighborhood in order to function appropriately. A requirement for matching is illustrated in transgene bypass experiments in which multimerized binding sites for specific architectural proteins are paired with themselves or with each other[52]. Bypass was observed when multimerized dCTCF, Zw5 or Su(Hw) binding sites were paired with themselves; however, heterologous combinations (e.g. dCTCF sites with Su(Hw) sites) did not support bypass. A second feature is that pairing interactions between boundaries are typically orientation dependent For example, *scs* pairs with itself head-to-head, not head-to-tail [52].

If both blocking and bypass activities are intrinsic properties of fly boundaries then the BX-C boundaries themselves may facilitate contacts between the regulatory domains and their target genes (Fig 1C). Moreover, the fact that both blocking and bypass activity are non-autonomous (in that they depend on partner pairing) could potentially explain why heterologous *Fab-7* replacements like *gypsy* and *Mcp* behave anomalously while *Fab-8* functions appropriately. Several observations fit with this idea. There is an extensive region upstream of the *Abd-B* promoter that has been implicated in tethering the *Abd-B* regulatory domains to the promoter [53–56] and this region could play an important role in mediating bypass by boundaries associated with the distal *Abd-B* regulatory domains (*iab-5*, *iab-6*, *iab-7*). Included in this region is a promoter tethering element (PTE) that facilitates interactions between *iab* enhancers and the *Abd-B* promoter in transgene assays [57,58]. Just beyond the PTE is a boundary-like element, *AB-I*. In transgene assays *AB-I* mediates bypass when combined with either *Fab-7* or *Fab-8*. In contrast, a combination between *AB-I* and *Mcp* fails to support bypass [59,60]. The ability of both *Fab-7* and *Fab-8* to pair with *AB-I* is recapitulated in *Fab-7* replacement experiments. Unlike *Mcp*, *Fab-8* has both blocking and bypass activity when inserted in place of *Fab-7* [30]. Moreover, its’ bypass but not blocking activity is orientation-dependent. When inserted in the same orientation as the endogenous *Fab-8* boundary, it mediates blocking and bypass, while it does not support bypass when inserted in the opposite orientation.

In the studies reported here we have tested this model by replacing the endogenous *Mcp* boundary with heterologous boundaries. *Mcp* defines the border between the set of regulatory domains that control *abd-A* and those that control *Abd-B* expression (Fig 1A). Unlike the boundaries that are within the *Abd-B* regulatory domain (e.g. *Fab-7* or *Fab-8*), *Mcp* is not located between a regulatory domain and its target gene. Instead, it defines the boundary between regulatory domains that target *abd-A* and those that target *Abd-B*. For this reason, we expected that it does not need bypass activity. Consistent with this expectation, we find that multimerized dCTCF binding sites fully substitute for *Mcp*. A different result is obtained for the *Abd-B*-associated boundaries, *Fab-7* and *Fab-8*. Both boundaries are (for the most part) able to block crosstalk between the *abd-A* regulatory domain *iab-4*, which specifies A4 (PS9) and the *Abd-B* regulatory domain *iab-5*, which specifies A5 (PS10). Their blocking activity is orientation independent. However, in spite of blocking crosstalk, these replacements still inappropriately induce *Abd-B* expression in A4 (PS9), causing the misspecification of this segment. For the *Fab-7* replacements, this occurred in both orientations, while for the *Fab-8* replacement ectopic induction was only observed when it was inserted in the same orientation as the endogenous *Fab-8* boundary. We present evidence showing that the boundary replacements activate the *Abd-B* gene in A4 (PS9) by inappropriately targeting the *iab-4* domain to the *Abd-B* promoter. In addition to altering the specification of A4 (PS9), the *Fab-7* replacements induce novel transformations of A5 and A6. These findings indicate that when *Fab-7* is inserted into the BX-C in place of *Mcp*, it perturbs *Abd-B* regulation in several segments besides PS9.

## Results

### Substitution of *Mcp* by an *attP* integration site in the BX-C

The *Mcp* boundary is defined by 340 bp core sequence that has enhancer blocking activity in transgene assays [36] and blocks crosstalk between *iab-6* and *iab-7* when substituted for *Fab-7* [31]. Located just distal to the boundary is a PRE that negatively regulates the activity of the *iab-5* enhancers [61]. We used CRISPR to delete a 789 bp DNA segment including the *Mcp* boundary and the PRE and replace it with an *eGFP* reporter flanked by two *attP* sites (*Mcp*^*attP*^) (S1 Fig). The presence of two *attP* sites in opposite orientation allows integration of different DNA fragments by recombination mediated cassette exchange (RMCE; [62]) using the *phiC31* integration system [63].

### Multimerized dCTCF sites substitute for *Mcp*

**Fig 2:**
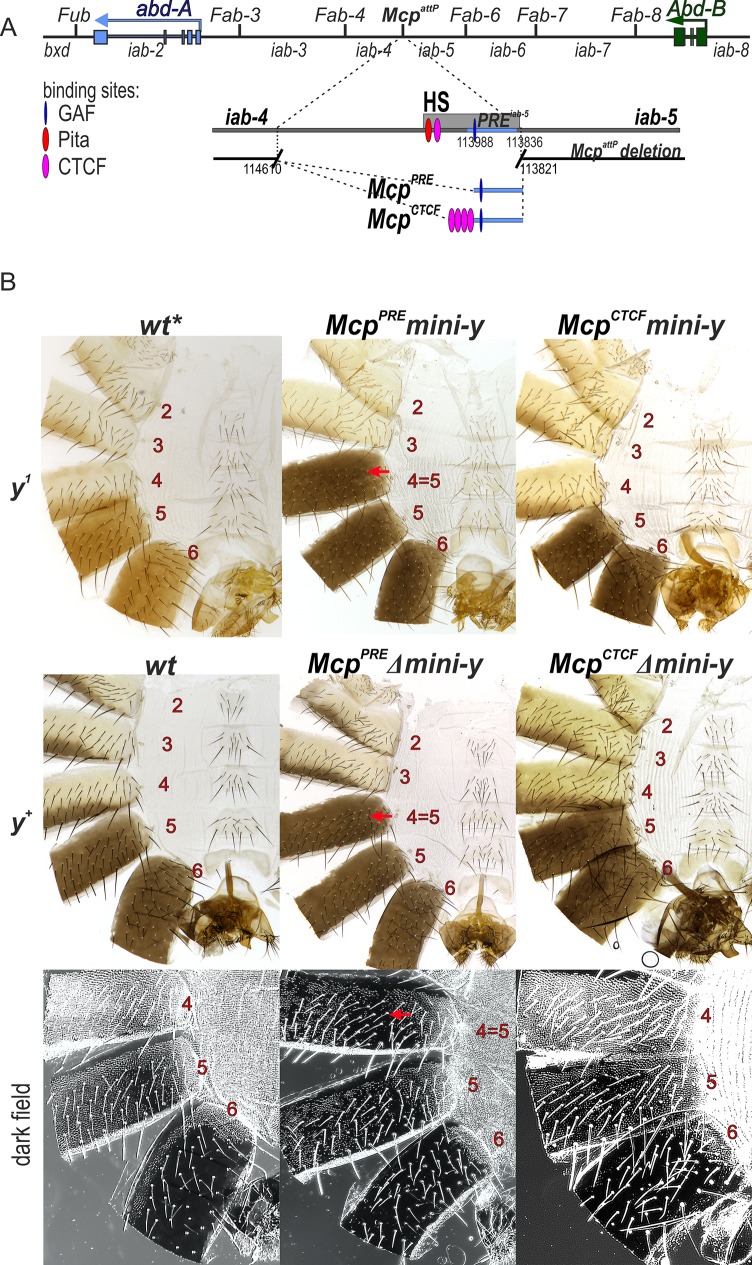
The CTCF sites block crosstalk between the *iab-4* and *iab-5* domains. (A) Molecular maps of the *Mcp* boundary. The coordinates of the *Mcp*^*attP*^ deletion and *Mcp*^*PRE*^ *Mcp*^*CTCF*^ replacement fragments according to complete sequence of BX-C in SEQ89E numbering [4] are shown below. DNAse hypersensitive site is shown as a light gray box above the coordinate bar. Binding sites for GAF, Pita and dCTCF are indicated by blue, red and purple ovals, respectively. PRE element from *iab-5* is marked as a blue stripe. Replacement fragments are shown below. wt* indicate wt with respect to *Mcp*. (B) The cuticle preparations of *wt*, *Mcp*^*PRE*^ and *Mcp*^*CTCF*^ males. The morphology of the 2^nd^ to 6^th^ abdominal segments is shown. Abnormalities in segment phenotype are shown by the red arrows. The localization of trichomes on the 4th to 6th abdominal tergites are shown in dark field.

The *Mcp* boundary marks the division between the set of regulatory domains that control the *abd-A* and *Abd-B* genes (Fig 1A). The *iab-4* domain is just proximal to *Mcp*, and it directs *abd-A* expression in PS9. The *iab-5* domain is on the distal side and it regulates *Abd-B* in PS10. A boundary in this position would be needed to block crosstalk between *iab-4* and *iab-5*; however, neither of these domains would require the intervening boundary to have bypass activity. On the proximal side, *iab-4* must bypass the putative *Fab-3* and *Fab-4* boundaries in order to activate the *abd-A* promoter, while on the distal side, *iab-5* must bypass *Fab-6*, *Fab-7* and *Fab-8* in order to activate *Abd-B*. If this expectation is correct, a generic boundary that has blocking activity but is unable to direct *iab-4* to the *abd-A* promoter or *iab-5* to the *Abd-B* promoter should be able to substitute for *Mcp*. To test this prediction (Fig 2), we introduced either the *iab-5* PRE itself (*Mcp*^*PRE*^) or the PRE in combination with four dCTCF sites (*Mcp*^*CTCF*^). In *Fab-7* replacement experiments four dCTCF sites in combination with the *iab-7* PRE blocked crosstalk between the *iab-6* and *iab-7* domains, but did not allow the *iab-6* domain to regulate *Abd-B* expression in PS11 [30].

*Abd-B* is a master regulator of pigmentation in the male abdominal A5 and A6 segments and it controls the expression of the *yellow* and *tan* genes which are involved in melanin synthesis [64–66]. In flies carrying the null *y*^*1*^ allele, the *tan* gene is still expressed and the pigmentation in A5 and A6 is light brown-yellow not black [66,67]. In order to be able to recover recombinants and also to monitor the blocking activity of the replacement sequence and the *on*/*off* state of the *iab-5* domain, we used a *y*^*1*^ genetic background and included a minimal *yellow* (*mini-y*) reporter in our *Mcp* replacement construct (S1 Fig). The *mini-y* reporter consists of the cDNA and the 340 bp *yellow* promoter and lacks the wing, body and bristle enhancers of the endogenous *yellow* gene. As a result, activity of the *mini-y* reporter depends upon proximity to nearby enhancers. Expression of the *mini-y* reporter was examined in the *y*^*1*^ background.

Based on previous studies [5,21,68], the expression of this reporter should be determined by the activity state of the *iab-5* domain. When *iab-5* is shutoff by Pc-G dependent silencing in PS9 and more anterior parasegments, the *mini*-*y* reporter will also be silenced. When *iab-5* is turned on in PS10 and more posterior parasegments, the *mini*-*y* reporter will be expressed. This parasegment-specific regulation of the reporter activity will be reflected in the segmental pattern of black melanin pigmentation in the adult cuticle. In replacements in which blocking activity is compromised, *mini*-*y* will be expressed in PS9 and in adults the A4 tergite will be black, just like the A5 and A6 tergites. In contrast, in replacements that have blocking activity *mini-y* will be silenced in PS9, but active in PS10 and PS11. In this case, A5 and A6 will have black pigmentation, while the stripe of pigmentation along the posterior edge of the A4 tergite will be light yellow brown, as only the *tan* gene will contribute to pigmentation in this segment.

When we replaced the *Mcp* deletion by the *iab-5* PRE alone (*Mcp*^*PRE*^) the *mini*-*y* reporter was active not only in A5 (PS10) and more posterior segments, but also in A4 (PS9).

**Fig 3:**
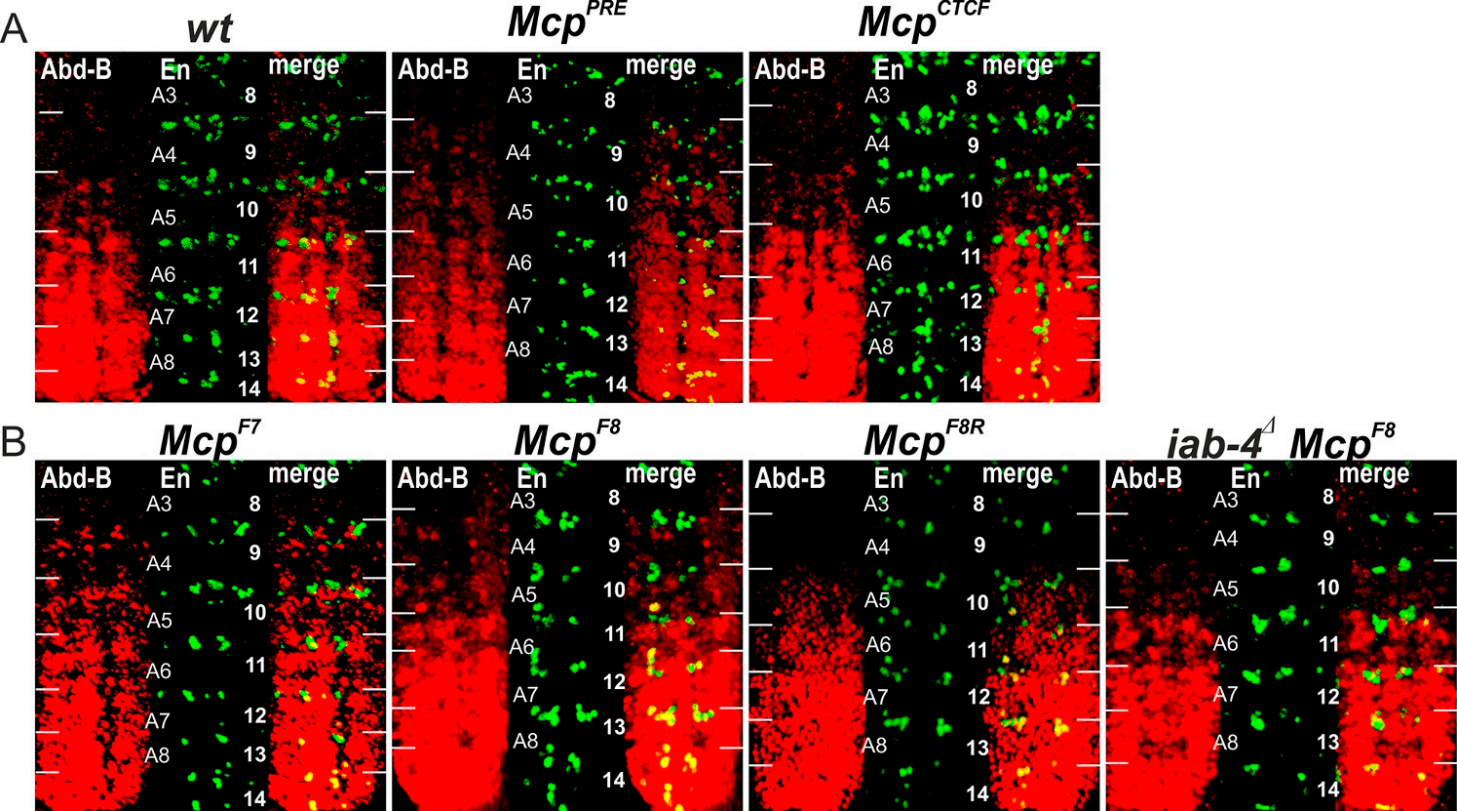
Expression of *Abd-B* in *Mcp* replacement embryos. (A) *Abd-B* expression in *wt*, *Mcp*^*PRE*^ and *Mcp*^*CTCF*^ embryos. (B) *Abd-B* expression in *Mcp*^*F7*^, *Mcp*^*F8*^, *Mcp*^*F8R*^ and *iab-4*^*Δ*^ *Mcp*^*F8*^ embryos. Each panel shows an image of the embryonic CNS of stage 14 embryos stained with antibodies to Abd-B (red) and Engrailed (En, green). En is used to mark the anterior boundary of each parasegment. White horizontal bars approximately delimit parasegment/segment boundaries. Parasegments numbered from 9 to 14 on the right side of the panels; approximate positions of segments are shown on the left side of the wild type (wt) panel and marked A4 to A8. The wild type expression pattern of *Abd-B* in the embryonic CNS is characterized by a stepwise gradient of increasing protein level from PS10 to PS14. The *Mcp*^*F8*^ or *Mcp*^*F7*^ embryos have similar low *Abd-B* expression in PS9 and PS10. The *Abd-B* expression in PS9 is absent in *iab-4*^*Δ*^ *Mcp*^*F8*^ and *Mcp*^*F8R*^ embryos.

As shown in Fig 2, the pigmentation in A4 is black like that in A5 indicating that the reporter is expressed in both segments (Fig 2). This finding shows that, similar to classical *Mcp* deletions, the *Mcp*^*PRE*^ replacement does not have blocking activity. In these *Mcp* deletions *iab-5* is ectopically activated in PS9 by the *iab-4* initiator. It then drives *Abd-B* expression in PS9 resulting in a gain-of-function (GOF) transformation of parasegment identity from PS9 to PS10. We used two approaches to test whether this was true for the *Mcp*^*PRE*^ replacement. In the first, we excised the *mini-y* reporter and introduced an X chromosome with a wild type *yellow* (*y*^*+*^) gene. Since *Abd-B* directly regulates *y*^*+*^ expression in the abdomen [65,67], a transformation of PS9 into PS10 should be accompanied by a PS10-like pattern of pigmentation. Fig 2 shows that this is the case. We also examined the pattern of Abd-B protein expression in the embryonic CNS. In wild type embryos Abd-B is not expressed is PS9, while it is expressed at low levels in PS10. As shown in Fig 3A, similar levels of Abd-B protein are detected in PS9 and PS10 in the *Mcp*^*PRE*^ replacement.

As predicted, a quite different result is obtained when we combined the *iab-5* PRE with multimerized dCTCF sites. Expression of the *mini-y* reporter in the *Mcp*^*CTCF*^ replacement was restricted to A5 (PS10) and A6 (PS11) as would be expected if the multimerized dCTCF sites block crosstalk between the *iab-4* and *iab-5* domains so that *iab-5* is silenced by Pc-G factors in PS9 (Fig 2). The same pigmentation pattern is observed for the endogenous *yellow* in the Δ*mini-y* derivative of *Mcp*^*CTCF*^, indicating that *Abd-B* is not turned on ectopically in PS9. This conclusion is confirmed by antibody staining experiments (Fig 3A). Thus, unlike replacements of *Fab-7*, a generic boundary can fully substitute for *Mcp*.

### Substitution of *Mcp* for *Fab-7* disrupts *Abd-B* regulation in parasegments PS9, PS10 and PS11

**Fig 4:**
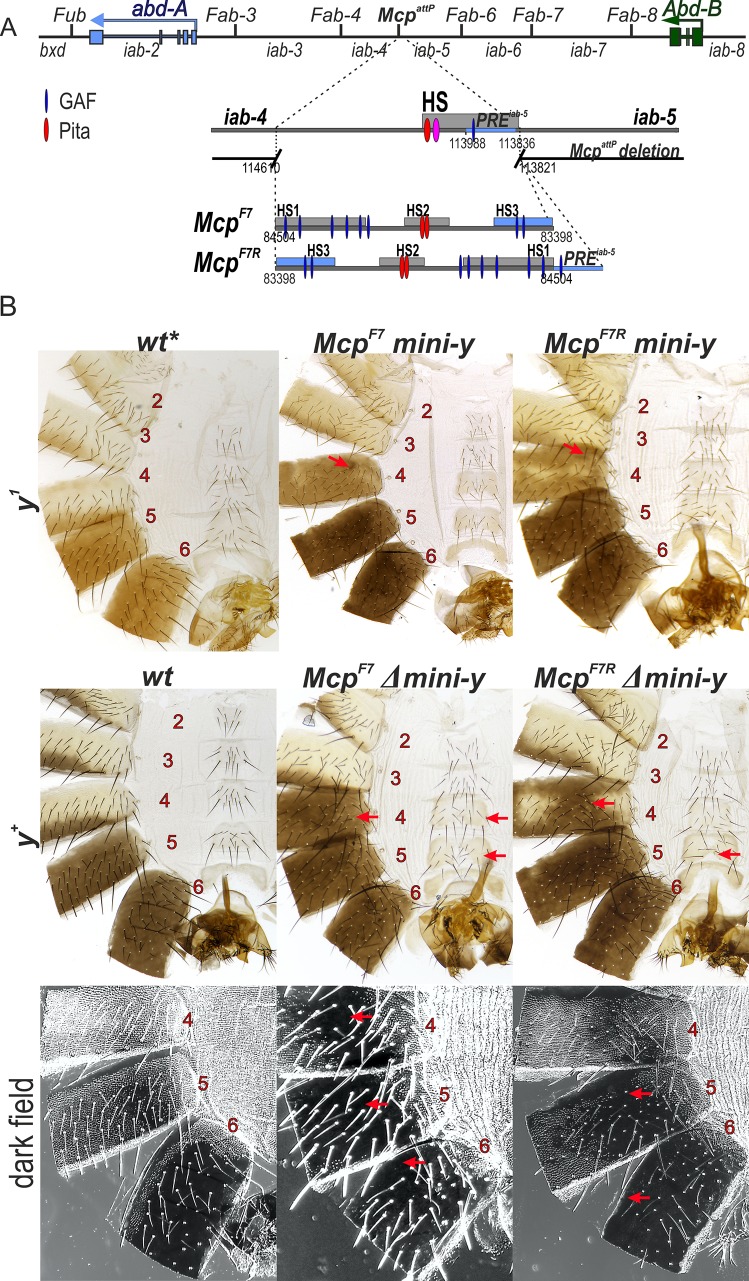
*Mcp*^*F7*^ and *Mcp*^*F7R*^ support *Abd-B* activation in the A4 segment. (A) Schematic representation of the *Fab-7* boundary. The 1.1 kb *Fab-7* replacement consists of HS1, HS2 and HS3 (*iab-7* PRE) regions (shown as blue box). (B) Morphology of the 2^nd^ to 6^th^ abdominal segments in *Mcp*^*F7*^ and *Mcp*^*F7R*^ males. Other designations are as in Fig 2.

We next tested whether the *Fab-7* boundary can substitute for *Mcp*. The *Fab-7* region consists of a minor (HS*) and three major (HS1, HS2 and HS3) nuclease hypersensitive sequences [17,21,22,41,42]. Unlike *Mcp* or other known or suspected boundaries in BX-C, dCTCF does not bind to *Fab-7* [33,69]. Instead, *Fab-7* boundary function depends upon two BEN domain protein complexes, Elba and Insensitive, the C_2_H_2_ zinc finger protein Pita, and a large multiprotein complex, called the LBC [31,70–73]. In addition to a boundary function, the HS3 sequence can also function as a PRE (*iab-7* PRE; [73,74]. In previous studies, we found that a combination of HS1+HS2+HS3 can functionally substitute for the complete *Fab-7* boundary *in vivo* and we used this sequence (named for simplicity *F7*) for the *Mcp* replacements (Fig 4). Although *Fab-7* has only limited orientation dependence in its endogenous context [30,73], we inserted the HS1+HS2+HS3 sequence in both the forward (same as endogenous *Fab-7*) and reverse orientations in the *Mcp* replacement platform as indicated in Fig 4. The phenotypic effects of the *Fab-7* replacement inserted in the forward orientation, *Mcp*^*F7*^, are considered first.

Like the *Mcp*^*CTCF*^ replacement, the *mini-y* reporter is turned on in A5 (PS10) and A6 (PS11) in *Mcp*^*F7*^ males, and the tergites in both of these segments are black. However, *Mcp*^*F7*^ differs in two respects from *Mcp*^*CTCF*^. First, there are one or two small patches of darkly pigmented cuticle in the A4 tergite (marked by the arrow). These patches are variable and appear to be clonal in origin. This finding indicates that the blocking activity of *Mcp*^*F7*^ is incomplete, and that the *mini-y* reporter and thus the *iab-5* domain is ectopically activated by the *iab-4* domain in a small number of PS9 cells. Second, instead of a stripe of yellow-brown pigmentation along the posterior margin, nearly the entire A4 tergite is covered in yellow-brown pigmentation. This pattern of pigmentation is not observed in A4 in *y*^*1*^ males carrying the *Mcp*^*CTCF*^ replacement and the *mini-y* reporter (Fig 2) or for that matter in control wild type *y*^*1*^ males (see Fig 4). The presence of the yellow-brown pigmentation throughout most of the A4 tergite suggests that cells in this segment (PS9) are not properly specified. This is the case. When the *mini-y* reporter was excised and replaced by the endogenous X-linked *y*^*+*^ gene, the A4 tergite has a black pigmentation like A5 and A6 (Fig 4). Since expression of the *yellow* gene is controlled by *Abd-B*, this observation indicates that *Abd-B* must be ectopically activated throughout A4. Antibody staining experiments of the CNS in *Mcp*^*F7*^ embryos indicate that this inference is correct (Fig 3B).

A simple interpretation of these findings is that *Mcp*^*F7*^ is unable to block crosstalk between *iab-4* and *iab-5* and, as a result, *iab-5* is ectopically activated in PS9 cells and inappropriately drives *Abd-B* expression. However, such an interpretation is inconsistent with the expression pattern of the *mini-y* reporter; it is only activated in small clones in the A4 tergite and not in the entire A4 tergite. By way of comparison, the dark black pigmentation generated by the reporter in *Mcp*^*PRE*^, which has no boundary activity, is clearly quite different from the yellow-brown pigmentation observed for the reporter in *Mcp*^*F7*^. In this respect, *Mcp*^*F7*^ resembles *Mcp*^*CTCF*^ in that the *iab-5* domain must be shut off by Pc-G silencing in (most) PS9 cells. This would imply that the *iab-4* regulatory domain (or one of the other *abd-A* domains that is turned on in PS9 cells) must be responsible for ectopically activating *Abd-B* expression in PS9. Moreover, this would mean that the mechanism underlying the misspecification of A4 (PS9) in the *Mcp*^*F7*^ replacement differs from that in *Mcp*^*PRE*^ or *Mcp*^*1*^ where *iab-5* is not properly silenced in PS9 cells.

There are other abnormalities in *Mcp*^*F7*^ replacement indicating that it has complicated and novel effect on *Abd-B* expression. In wild type males, the A6 sternite has a banana shape and no bristles, while the A5 and A4 sternites resemble isosceles trapezoids and are covered with bristles. While the A4 and A5 sternites in *Mcp*^*F7*^ males still have bristles, they are split into two connected lobes that resemble the banana shape of the A6 sternite. These morphological abnormalities indicate that the *Fab-7* replacement induces a weak GOF transformation of both A4 (PS9) and A5 (PS10) towards an A6 (PS11) identity. This type of transformation is not observed in *Mcp* boundary deletions, nor it is observed in the *Mcp*^*PRE*^ replacement.

Further evidence of A4/A5→A6 transformation can be seen in the pattern of trichome hairs in the tergites. In wild type flies, the A4 and A5 tergites are covered with trichomes, while trichomes are only found along the anterior and ventral margins of the A6 tergite (see darkfield image in Fig 4). In the *Mcp*^*F7*^ replacement, there are large regions of the A4 and A5 tergite that are devoid of trichomes. There are even anomalies in A6: the band of trichomes along the anterior margin is absent. Similar alterations in cuticular phenotypes are observed in *Mcp*^*F7*^ females (S2 Fig). These findings indicate that the normal regulation of *Abd-B* is disrupted in several parasegments when *Mcp* is replaced by the *Fab-7* boundary.

In its endogenous context, the functioning of *Fab-7* is weakly orientation dependent. For this reason, we anticipated that the reverse *Mcp* replacement, *Mcp*^*F7R*^, would give a similar though milder spectrum of phenotypic effects. Fig 4 shows that this is the case. In *y*^*+*^ background, large regions of the A4 tergite have a black pigmentation like A5 and A6. The ectopic activation appears to be weaker than in the *Mcp*^*F7*^ replacement as there are regions in A4 in which the endogenous *yellow* gene is not turned on. Also, and unlike *Mcp*^*F7*^, there are no bald patches in the A4 trichomes, while the sternite appears to have a normal isosceles trapezoid shape. However, the novel transformations seen in *Mcp*^*F7*^ in the more posterior segments A5 (PS10) and A6 (PS11) are still evident. The A5 tergite is not completely covered with trichomes, while the trichomes along the anterior margin of A6 are absent. The A5 sternite is also misshapen. Thus, like *Mcp*^*F7*^, introducing a reversed *Fab-7* boundary in place of *Mcp* disrupts *Abd-B* regulation in PS9 and also in other parasegments. Since the pattern of *mini-y* expression in *Mcp*^*F7R*^ indicates that the *iab-5* domain is silenced in (most) PS9 cells, the *iab-5* regulatory domain can’t be driving *Abd-B* expression in this parasegment. Instead, misexpression of *Abd-B* in PS9 is likely driven by the *iab-4* domain. This possibility will be considered further below.

### The *Fab-8* boundary displays orientation-dependent effects on ectopic activation of *Abd-B* in the A4 abdominal segment

In previous *Fab-7* replacement experiments we found that a 337 bp fragment (*F8*^*337*^) spanning the *Fab-8* boundary nuclease hypersensitive site is sufficient to fully rescue a *Fab-7* boundary deletion [30]. In the direct (forward) orientation this fragment not only blocks crosstalk but also supports bypass. However, when the orientation of the *Fab-8* boundary is reversed, bypass activity is lost, while blocking is unaffected. Since *F8*^*337*^ appears to have full boundary function, we inserted this fragment in both orientations next to the *iab-5 PRE* in the *Mcp* deletion (*Mcp*^*F8*^ and *Mcp*^*F8R*^).

**Fig 5:**
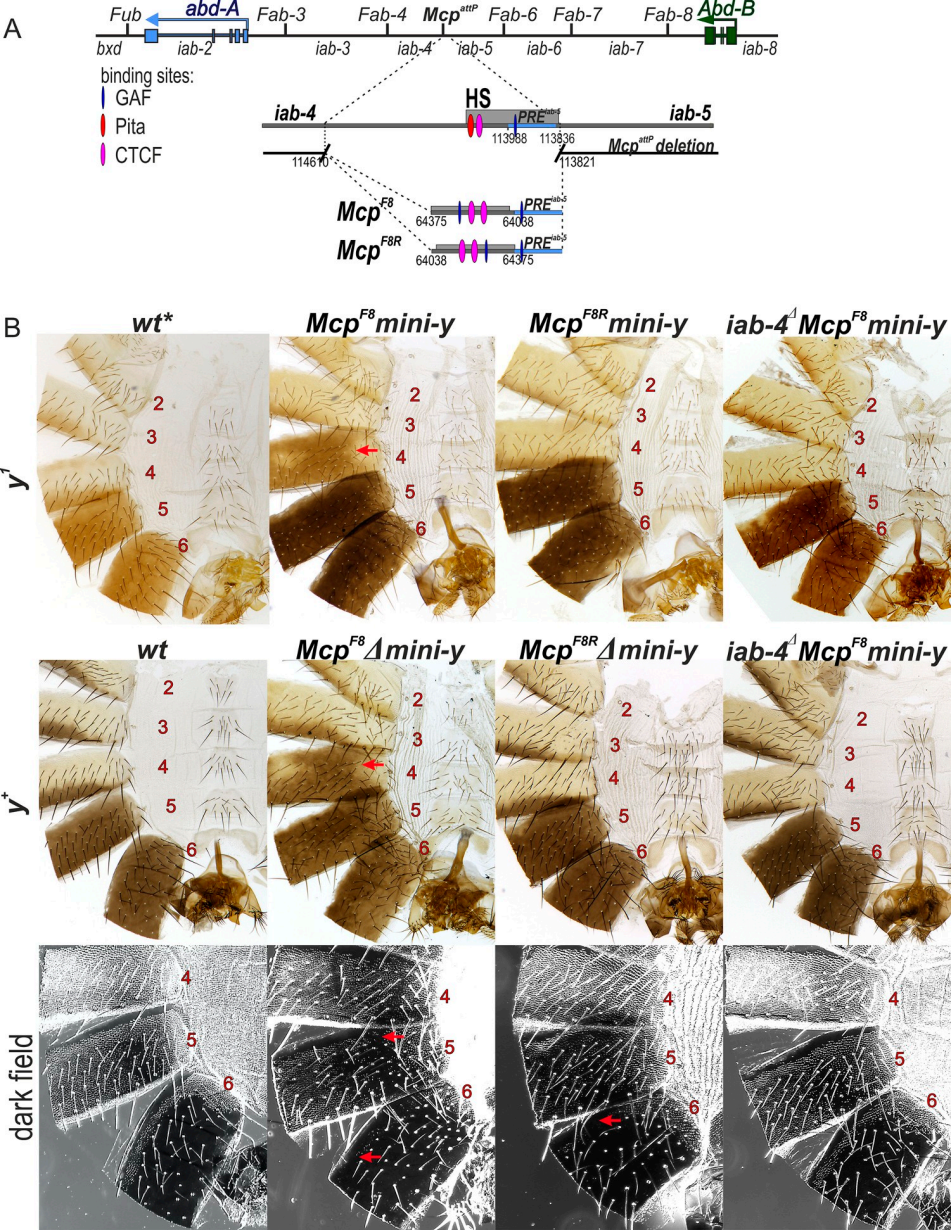
Activation of *Abd-B* by the *iab-4* enhancer depends on the orientation of the *Fab-8* insulator in *Mcp*^*F8*^ and *Mcp*^*F8R*^ mutants. (A) Molecular maps of the *Fab-8* boundary and *F8*^*337*^. The *Fab-8* insulator is shown as a horizontal bar. The proximal and distal endpoints of the *Fab-8* fragments are shown below. For other designations see Fig 2. (B) Morphology of the 2^nd^ to 6^th^ abdominal segments in insulator in *Mcp*^*F8*^, *Mcp*^*F8R*^ and *iab-4*^*Δ*^ *Mcp*^*F8*^ males. Other designations are as in Fig 2.

The effects of the *Fab-8* replacement in the reverse orientation, *Mcp*^*F8R*^, will be considered first. Like the *Mcp*^*CTCF*^ replacement, *Mcp*^*F8R*^ blocks crosstalk between *iab-4* and *iab-5* and the *mini-y* reporter is off in A4 (Fig 5). The fact that *mini-y* is not expressed in PS9 also means that *iab-5* is silenced as it should be in PS9 cells. After the deletion of the *mini-y* reporter and introducing a wild type *y*^*+*^ allele, the pigmentation in the adult male abdomen is equivalent to that in wild type flies. The morphological features of *Mcp*^*F8R*^ tergites and sternites also resemble those in wild type flies or the *Mcp*^*CTCF*^ replacement and there is no indication of the other abdominal transformations seen in the *Fab-7* replacements. Consistent with the phenotype of the adult cuticle, the pattern of *Abd-B* expression in the embryonic CNS resembles wild type (Fig 3B). Thus, the *Mcp*^*F8R*^ replacement fully substitutes for the endogenous *Mcp* boundary.

A different result is obtained when the *F8*^*337*^ sequence is inserted in its normal forward orientation. Like the reverse orientation *Mcp*^*F8R*^, *Mcp*^*F8*^ efficiently blocks crosstalk between *iab-4* and *iab-5* and the *mini-y* reporter is not activated in A4 (PS9). On the other hand, like the *Fab-7* replacements (*Mcp*^*F7*^ and *Mcp*^*F7R*^) most of the A4 tergite is covered in a yellow brown pigmentation instead of the normal stripe of yellow brown pigmentation along the posterior margin of the tergite that is seen in *y*^*1*^ males. Moreover, when the reporter is excised and the *y*^*1*^ allele replaced by the wild type *y*^*+*^ gene, nearly the entire A4 tergite is black. Consistent with the induction of *y*^*+*^ expression in A4, *Abd-B* is active in PS9 in the embryonic CNS (Fig 3B). The GOF transformation of A4 (PS9)→A5 (PS10) is not the only anomaly in *Mcp*^*F8*^ flies. While there does not seem to be any misspecification of the tergite or sternites in A5 (PS10), the line of trichomes along the anterior margin of the A6 tergite is disrupted or absent altogether indicating that there are some abnormalities in the temporal and/or special pattern of *Abd-B* expression in PS11.

### Ectopic expression of *Abd-B* in A4 (PS9) requires a functional *iab-4* domain

In the *Fab-7* replacement experiments, the relative orientation of the *Fab-8* boundary was thought to be important because it determined whether the chromatin loops formed between the replacement boundary and the *AB-I* element and/or the PTE sequence upstream of the *Abd-B* transcription start site were circle loops or stem loops [30,75]. In the forward orientation circle loops are expected to be formed and in this configuration, the downstream *iab-5* regulatory domain is brought into close proximity with the *Abd-B* promoter. In the reverse orientation, *iab-6* and *iab-7* are predicted to form stem loops, and this configuration would tend to isolate the *iab-5* regulatory domain from the *Abd-B* promoter.

It seemed possible that a similar mechanism might be in play in the *Fab-8* replacements of *Mcp*. In the forward orientation (*Mcp*^*F8*^), the *iab-4* regulatory domain would be brought into close proximity to the *Abd-B* gene, activating its ectopic expression in A4 (PS9). In the opposite orientation, the spatial relationship between the *iab-4* domain and the *Abd-B* promoter would not be conducive for activation. In this case, *Abd-B* would be off in A4 (PS9). A strong prediction of this model is that the inappropriate activation of *Abd-B* in PS9 in the *Mcp*^*F8*^ replacement should depend on a functional *iab-4* domain.

To test this prediction, we used CRISPR (see S3 Fig) to delete a 4,401 bp sequence (*iab-4*^*Δ*^) that spans the putative *iab-4* initiation element in flies carrying the *Mcp*^*F8*^ replacement. The *iab-4*^*Δ*^ sequence was selected based on the clustering of multiple binding sites for transcription factors controlling segmentation of the embryo. [76]. Fig 5 shows that the ectopic activation of *y*^*+*^ in A4 in *Mcp*^*F8*^ flies was eliminated by the *iab-4*^*Δ*^ deletion. Moreover, *Abd-B* was not activated in A4 (PS9) in the embryonic CNS of *iab-4*^*Δ*^ *Mcp*^*F8*^ embryos (Fig 3B). Interestingly, the loss of trichomes along the anterior margin of the A6 tergite in *Mcp*^*F8*^ also seemed to depend on a functional *iab-4* domain. As can be seen in Fig 5, the trichome pattern in the A6 tergite of *iab-4*^*Δ*^ *Mcp*^*F8*^ flies resembled that of wild type.

## Discussion

Boundaries flanking the *Abd-B* regulatory domains must block crosstalk between adjacent regulatory domains but at the same time allow more distal domains to jump over one or more intervening boundaries and activate *Abd-B* expression. While several models have been advanced to account for these two paradoxical activities, replacement experiments argued that both must be intrinsic properties of the *Abd-B* boundaries. Thus *Fab-7* and *Fab-8* have blocking and bypass activities in *Fab-7* replacement experiments, while heterologous boundaries including multimerized dCTCF sites and *Mcp* from BX-C do not. One idea is that *Fab-7* and *Fab-8* are simply “permissive” for bypass. They allow bypass to occur, while boundaries like multimerized dCTCF or *Mcp* are not permissive in the context of *Fab-7*. Another is that they actively facilitate bypass by directing the distal *Abd-B* regulatory domains to the *Abd-B* promoter. Potentially consistent with an “active” mechanism that involves boundary pairing interactions, the bypass activity of *Fab-8* and to a lesser extent *Fab-7* is orientation dependent.

In the studies reported here we have tested these two models further. For this purpose we used the *Mcp* boundary for *in situ* replacement experiments. *Mcp* defines the border between the regulatory domains that control expression of *abd-A* and *Abd-B*. In this location, it is required to block crosstalk between the flanking domains *iab-4* and *iab-5*, but it does not need to mediate bypass. In this respect, it differs from the boundaries that are located within the set of regulatory domains that control either *abd-A* or *Abd-B*, as these boundaries must have both activities. If bypass were simply passive, insertion of a “permissive” *Fab-7* or *Fab-8* boundary in either orientation in place of *Mcp* would be no different from insertion of a generic “non-permissive” boundary such as multimerized dCTCF sites. Assuming that *Fab-7* and *Fab-8* can block crosstalk out of context, they should fully substitute for *Mcp*. In contrast, if bypass in the normal context involves an active mechanism in which more distal regulatory domains are brought to the *Abd-B* promoter, then *Fab-7* and *Fab-8* replacements might also be able to bring *iab-4* to the *Abd-B* promoter in a configuration that activates transcription. If they do so, then this process would be expected to show the same orientation dependence as is observed for bypass of the *Abd-B* regulatory domains in *Fab-7* replacements.

Consistent with the idea that a boundary located at the border between the domains that regulate *abd-A* and *Abd-B* need not have bypass activity, we found that multimerized binding sites for the dCTCF protein fully substitute for *Mcp*. Like the multimerized dCTCF sites, *Fab-7* and *Fab-8* are also able to block crosstalk between *iab-4* and *iab-5*. In the case of *Fab-7*, its’ blocking activity is incomplete and there are small clones of cells in which the *mini-y* reporter is activated in A4. In contrast, the blocking activity of *Fab-8* is comparable to the multimerized dCTCF sites and the *mini-y* reporter is off throughout A4. One plausible reason for this difference is that *Mcp* and the boundaries flanking *Mcp* (*Fab-4* and *Fab-6*) utilize dCTCF as does *Fab-8*, while this architectural protein does not bind to *Fab-7* [33].

Importantly, in spite of their normal (or near normal) ability to block crosstalk, both boundaries still perturb *Abd-B* regulation. In the case of *Fab-8*, the misregulation of *Abd-B* is orientation dependent just like the bypass activity of this boundary when it is used to replace *Fab-7* [30]. When inserted in the reverse orientation, *Fab-8* behaves like multimerized dCTCF sites and it fully rescues the *Mcp* deletion. In contrast, when inserted in the forward orientation, *Fab-8* induces the expression of *Abd-B* in A4 (PS9), and the misspecification of this parasegment. Unlike classical *Mcp* deletions or the *Mcp*^*PRE*^ replacement described here, expression of the *Abd-B* gene in PS9 is driven by *iab-4*, not *iab-5*. This conclusion is supported by two lines of evidence. First, the *mini-y* reporter inserted in *iab-5* is off in PS9 cells indicating that *iab-5* is silenced by PcG factors as it should be in this parasegment. Second, the ectopic expression of *Abd-B* is eliminated when the *iab-4* regulatory domain is inactivated.

Our results, taken together with previous studies [30,59,60], support a model in which the chromatin loops formed by *Fab-8* inserted at *Mcp* in the forward orientation brings the enhancers in the *iab-4* regulatory domain in close proximity to the *Abd-B* promoter, leading to the activation of *Abd-B* in A4 (PS9). In contrast, when inserted in the opposite orientation, the topology of the chromatin loops formed by the ectopic *Fab-8* boundary are not compatible with productive interactions between *iab-4* and the *Abd-B* promoter. Moreover, it would appear that boundary bypass for the regulatory domains that control *Abd-B* expression is not a passive process in which the boundaries are simply permissive for interactions between the regulatory domains and the *Abd-B* promoter. Instead, it seems to be an active process in which the boundaries are responsible for bringing the regulatory domains into contact with the *Abd-B* gene. It also seems likely that bypass activity of *Fab-8* (and also *Fab-7*) may have a predisposed preference, namely it is targeted for interactions with the *Abd-B* gene. This idea would fit with transgene bypass experiments, which showed that both *Fab-7* and *Fab-8* interacted with an insulator like element upstream of the *Abd-B* promoter, *AB-I*, while the *Mcp* boundary didn’t [59,60].

Similar conclusions can be drawn from the induction of *Abd-B* expression in A4 (PS9) when *Fab-7* is inserted in place of *Mcp*. Like *Fab-8*, this boundary inappropriately targets the *iab-4* regulatory domain to *Abd-B*. Unlike *Fab-8*, *Abd-B* is ectopically activated when *Fab-7* is inserted in both the forward and reverse orientations. While the effects are milder in the reverse orientation, the lack of pronounced orientation dependence is consistent with experiments in which *Fab-7* was inserted at its endogenous location in the reverse orientation. Unlike *Fab-8* only very minor *iab-6* bypass defects were observed. In addition to the activation of *Abd-B* in A4 (PS9) the *Fab-7 Mcp* replacements also alter the pattern of *Abd-B* regulation in more posterior segments. In the forward orientation, A4 and A5 are transformed towards an A6 identity, while A6 is also misspecified. Similar though somewhat less severe effects are observed in these segments when *Fab-7* is inserted in the reverse orientation. At this point the mechanisms responsible for these novel phenotypic effects are uncertain. One possibility is that pairing interactions between the *Fab-7* insert and the endogenous *Fab-7* boundary disrupt the normal topological organization of the regulatory domains in a manner similar to that seen in boundary competition transgene assays [77]. An alternative possibility is that *Fab-7* targets *iab-4* to the *Abd-B* promoter not only in A4 (PS9) but also in cells in A5 (PS10) and A6 (PS11). In this model, *Abd-B* would be regulated not only by the domain that normally specifies the identity of the parasegment (e.g., *iab-5* in PS10), but also by interactions with *iab-4*. This dual regulation would increase the levels of *Abd-B*, giving the weak GOF phenotypes. Potentially consistent with this second model, inactivating *iab-4* in the *Mcp*^*F8*^ replacement not only rescues the A4 (PS9) GOF phenotypes but also suppresses the loss of anterior trichomes in the A6 tergite.

## Materials and methods

### Generation of *Mcp*^*attP*^ by *CRISPR/Cas9*-induced homologous recombination

The backbone of the recombination plasmid was designed *in silico* and contains several genetic elements in the following order: [*MCS5*]-[*attP*]-[*3xP3-EGFP-SV40polyA*]-[*attP*]-[*FRT*]-[*MCS3*]. This DNA fragment was synthesized and cloned into *pUC57* by Genewiz. The two multiple cloning sites *MCS5* and *MCS3* were used to clone homology arms into this plasmid. The orientations of the two *attP* sites are inverted relative to each other and serve as targets for *фC31*-mediated recombination mediated cassette exchange [62]. The *3xP3-EGFP* reporter [78] was introduced as a means to isolate positive recombination events. The *Flp*-recombinase target *FRT* [79] was included for the deletion of the selectable *mini-yellow* marker after recombination mediated cassette exchange.

Homology arms were PCR-amplified from *y w* genomic DNA using the following primers: CCTGCCGACTGAACGAATGC and ACGCCCTGATCCCGATACACATAC for the proximal arm (*iab-4* side; 3967 bp fragment), and GCGTTTGTGTGTAGTAAATGTATC and AAAGGCCAACAAAGAACACATGGACG for the distal arm (*iab-5* side; 4323 bp fragment). A successful homologous recombination event will generate a 789 bp deletion within the *Mcp* region (Genome Release R6.22: 3R:16’868’830–16’869’619; or complete sequence of BX-C: 113821–114610 [4]).

The recombination plasmid was injected into *y w vas-Cas9* embryos together with two gRNAs containing the following guides: GCTGGCTTTTACAGCATTTC and GCTTTGTTACCCCTGAAAAT. Concentrations were as described in Gratz et al.[80]. The injected embryos were grown to adulthood and crossed with *y w* partners. Among the few fertile crosses, one produced many larvae with a clear GFP-signal in the posterior part of their abdomens. This observation suggested that these animals had integrated the recombination plasmid and that the *3xP3-EGFP* reporter acts as an enhancer trap for *Abd-B* specific enhancers. GFP positive larvae were isolated and grown to adulthood. Emerging males showed the expected *Mcp* phenotype. Also, and as expected for a reporter located in the BX-C, no fluorescence signal could be detected in their eyes, indicating that the *3xP3-EGFP* reporter is silenced in eye cells where the *3xP3* promoter is usually active. The planned homologous recombination event could later be verified by PCR and sequencing. We will refer to it as *Mcp*^*attP*^.

12 *EGFP*- and *Mcp*-positive candidate males were individually crossed with *y w* virgins. Only 2 were fertile. The sterility of others may be caused by presence of *off*-targets as a frequent non-specific result of CRISPR/*Cas9* editing. Starting from the two fertile crosses, 2 independent balanced stocks could be obtained according to established crossing schemes. One of them was used to obtain a *y w M{vas-integrase}zh-2A; Mcp*^*attP*^*/TM3*,*Sb* stock for recombination mediated cassette exchange. Because of poor survival rates in injection experiments, the *Mcp*^*attP*^ chromosome was also temporarily combined with *Dp(3;3)P5*, *Sb* (*y w M{vas-integrase}zh-2A; Mcp*^*attP*^*/ Dp(3;3)P5*, *Sb*). By selection we obtained homozygous *Mcp*^*attP*^ line that was subsequently used for fly injections.

### Generation of *iab-4*^*Δ*^ by CRISPR/Cas9-induced homologous recombination

For generating dsDNA donors for homology-directed repair we used *pHD-DsRed* vector that was a gift from Kate O'Connor-Giles (Addgene plasmid # 51434). The final plasmid contains genetic elements in the following order: [*iab-4* proximal arm]-[*attP*]- [*lox*]- [*3xP3-dsRed-SV40polyA*]-[*lox*]- [*iab-4* distal arm]. Homology arms were PCR-amplified from *yw* genomic DNA using the following primers: TTT*GAATTC*TTCCAGACACGCATCGGG and AAA*CATATG*CTTGCTATCGACCCTCCTC for the proximal arm (846 bp fragment), and AAT*ACTAGT*CTCGGAAAGGGAAGAAGTTC and TAC*TCGAGC*CGCTAAAGGACGTTCTGC for the distal arm (835 bp fragment). A successful homologous recombination event will generate a 4401 bp deletion within the *iab-4* region (Genome Release R6.22: 3R:16,861,368..16,869,768; or complete sequence of BX-C [4]: 120073–115673).

Targets for *Cas9* were selected using “CRISPR optimal target finder”–the program from O'Connor-Giles Lab. The recombination plasmid was injected into *Mcp*^*F8*^ *vasa-Cas9* embryos together with two gRNAs containing the following guides: ATAGCAAGTAGGAGTGGAGT and GAACTTCTTCCCTTTCCGAGCGG. Concentrations were as described in Gratz et al. (2014). Injectees were grown to adulthood and crossed with *y w*; *TM6/MKRS* partners. Flies with clear dsRed-signal in eyes and the posterior part of their abdomens were selected into a new separate line. The successful integration of the recombination plasmid was verified by PCR.

### Cuticle preparations

3 day adult flies were collected in eppendorf tubes and stored in 70% ethanol at least 1 day. Then ethanol was replaced with 10% KOH and flies were heated at 70°C for 1–1.5h. After heating flies were washed with dH2O two times and heated again in dH2O for 45min. Then the digested flies were washed with 70% ethanol and stored in 70% ethanol. The abdomen cuticles were cut from the rest of the digested fly using fine tweezer and a needle of an insulin syringe and put in a droplet of glycerol on a glass slide. Then the abdomens were cut longitudinally on the dorsal side through all of the tergites with the syringe. To spread the cuticles flat on the slides cuts may be done between the tergites. Than the cuticles were flattened with a coverslip. Photographs in the bright or dark field were taken on the Nikon SMZ18 stereomicroscope using Nikon DS-Ri2 digital camera, processed with ImageJ 1.50c4 and Fiji bundle 2.0.0-rc-46.

### Embryo immunostaining

Primary antibodies were mouse monoclonal anti-Abd-B at 1:100 dilution (1A2E9, generated by S.Celniker, deposited to the Developmental Studies Hybridoma Bank) and polyclonal rabbit anti-Engrailed at 1:1000 dilution (a kind gift from Judith Kassis). Secondary antibodies were goat anti-mouse Alexa Fluor 647 (Molecular Probes) and anti-rabbit FITC conjugated (Jackson Research) at 1:2000 dilution. Stained embryos were mounted in the following solution: 23% glycerol, 10% Mowiol 4–88, 0.1M Tris-HCl pH 8.3. Images were acquired on Leica TCS SP-2 confocal microscope and processed using GIMP 2.8.16, ImageJ 1.50c4, Fiji bundle 2.0.0-rc-46.

## Supporting information

S1 Fig The strategy to create *Mcp* replacement lines. On the top: schematic representation of regulatory region of the *abd-A* and *Abd-B* genes (blue and green, respectively). The 789 bp *Mcp* region that was deleted (coordinates according to complete sequence of BX-C in SEQ89E numbering) and replaced by two *attP* sites for the integration of the tested constructs. *3xP3-eGFP* was used as a marker gene. *frt* site was used for excision of *yellow* maker gene. The plasmid that was injected into *Mcp*^*attP*^ line, contains two *attB* site for integration, *iab-5* PRE for restoring functional integrity of the *iab-5* domain, the *frt* site for excision of *yellow* gene, *lox* sites for excision of testing element. Testing elements were inserted just in front of *iab-5* PRE.
(TIF)

S2 Fig The abdominal cuticles of *wt*, *Mcp*^*F8*^, *Mcp*^*F8R*^, *Mcp*^*F7*^ and *Mcp*^*F7R*^ females. Morphology of the 2^nd^ to 6^th^ abdominal segments in *wt*, *Mcp*^*F8*^, *Mcp*^*F8R*^, *Mcp*^*F7*^ and *Mcp*^*F7R*^ females. The expression of *mini-y* (black pigment) is shown on the upper panel. Localization of trichomes on tergites is shown lower.
(TIF)

S3 Fig The strategy to create *iab-4* deletion. The scheme of the regulatory region in the distal part of the BX-C. Horizontal arrows represent transcripts for *abd-A* (blue) and *Abd-B* (green). The *iab-4* region was selected using FlyBase, based on the clustering of multiple binding sites for embryonic gap and pair-rule gene proteins. The screenshot show localization of the 4401 bp of *iab-4* deletion with R6 genome release coordinates. The coordinates of *iab-4* deletion according to complete sequence of BX-C (in SEQ89E numbering) are 120073–115673 (shown lower). The deletion was made using CRISPR/Cas9 strategy. Targets for Cas9 were selected using “CRISPR optimal target finder”–program from O'Connor-Giles Lab. Vector for generating dsDNA donors for homology-directed repair contains the visible marker *3xP3-DsRed*. *pHD-DsRed* was a gift from Kate O'Connor-Giles (Addgene plasmid # 51434). *dsRed* gene was using for selection of flies with *iab-4* deletion.
(TIF)
