# Supplementary figures and images for: Boundaries mediate long-distance interactions between enhancers and promoters in the Drosophila Bithorax complex
Source: PLoS Genet. 2018 Dec 12;14(12):e1007702. doi: 10.1371/journal.pgen.1007702 (PMC6306242; doi:10.1371/journal.pgen.1007702)

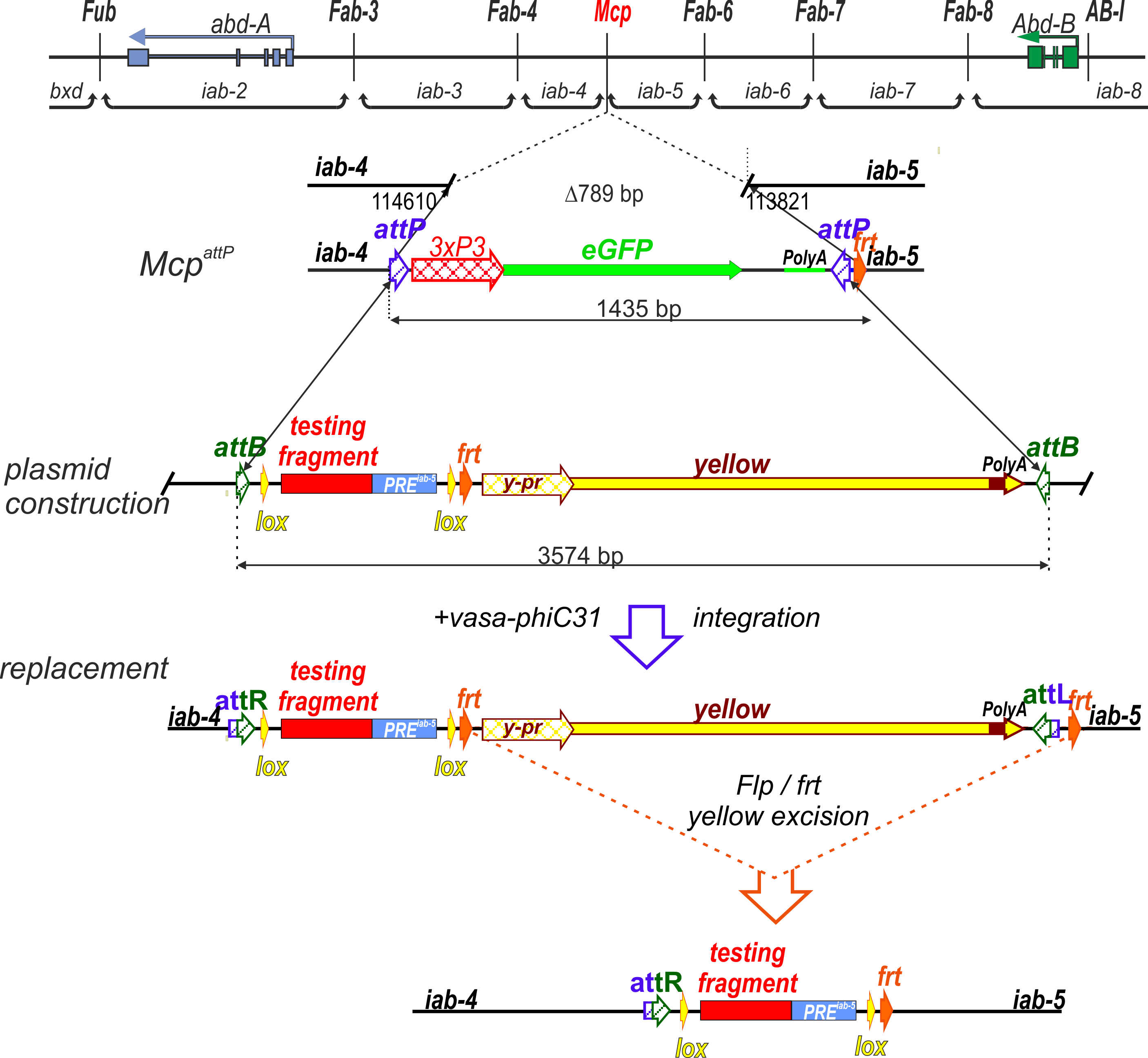

Supplement: S1 Fig — On the top: schematic representation of regulatory region of the abd-A and Abd-B genes (blue and green, respectively). The 789 bp Mcp region that was deleted (coordinates according to complete sequence of BX-C in SEQ89E numbering) and replaced by two attP sites for the integration of the tested constructs. 3xP3-eGFP was used as a marker gene. frt site was used for excision of yellow maker gene. The plasmid that was injected into McpattP line, contains two attB site for integration, iab-5 PRE for restoring functional integrity of the iab-5 domain, the frt site for excision of yellow gene, lox sites for excision of testing element. Testing elements were inserted just in front of iab-5 PRE. (TIF) [file pgen.1007702.s001.tif]

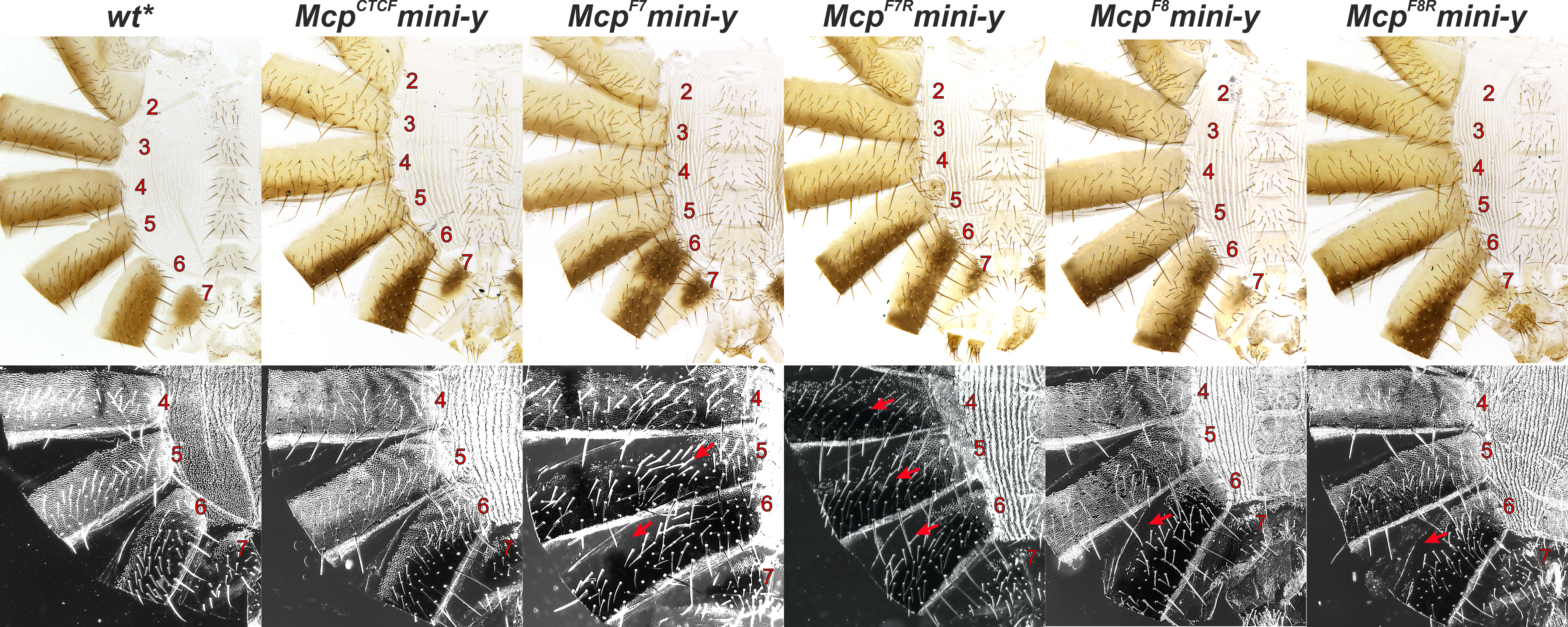

Supplement: S2 Fig — Morphology of the 2nd to 6th abdominal segments in wt, McpF8, McpF8R, McpF7 and McpF7R females. The expression of mini-y (black pigment) is shown on the upper panel. Localization of trichomes on tergites is shown lower. (TIF) [file pgen.1007702.s002.tif]

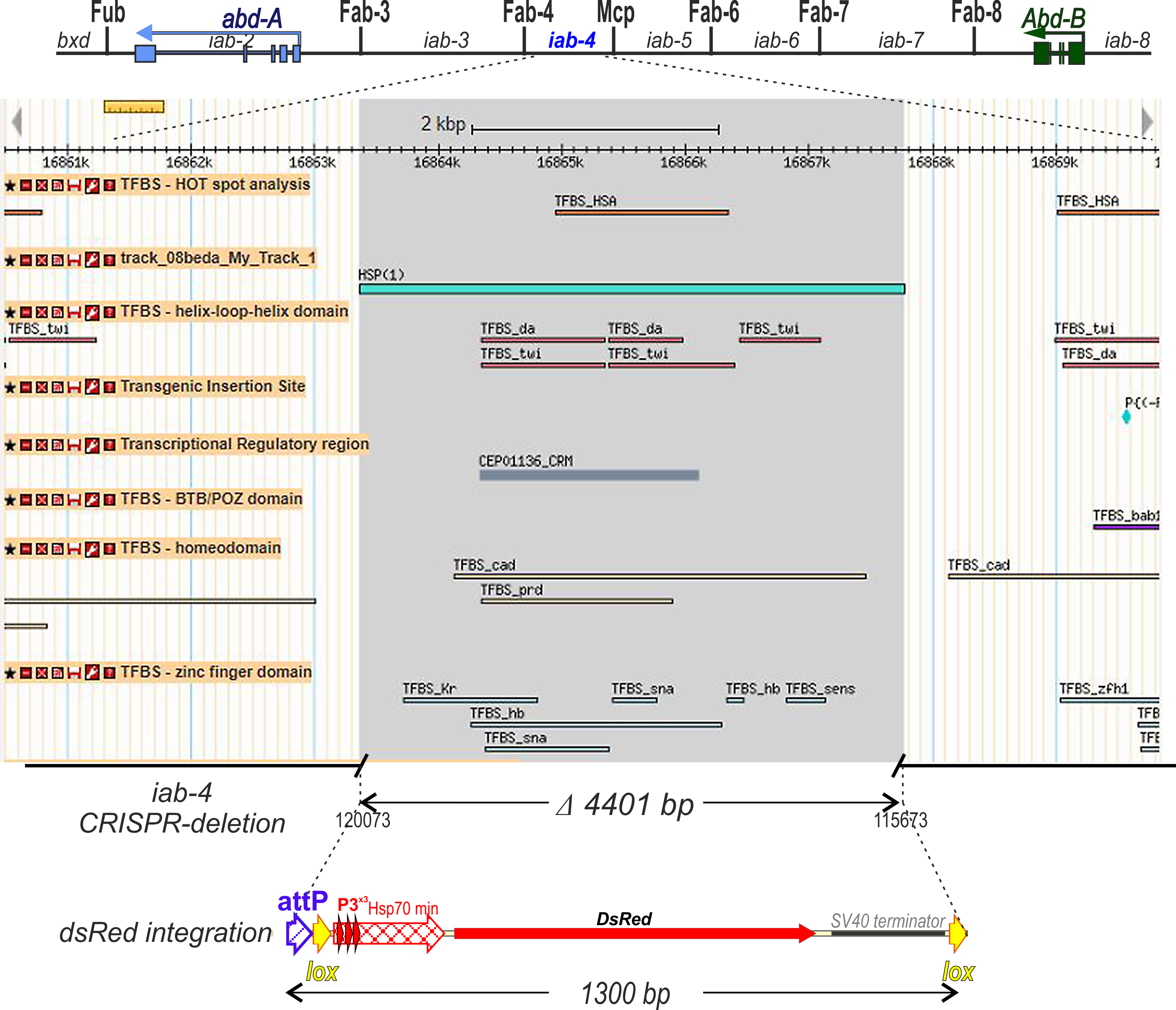

Supplement: S3 Fig — The scheme of the regulatory region in the distal part of the BX-C. Horizontal arrows represent transcripts for abd-A (blue) and Abd-B (green). The iab-4 region was selected using FlyBase, based on the clustering of multiple binding sites for embryonic gap and pair-rule gene proteins. The screenshot show localization of the 4401 bp of iab-4 deletion with R6 genome release coordinates. The coordinates of iab-4 deletion according to complete sequence of BX-C (in SEQ89E numbering) are 120073–115673 (shown lower). The deletion was made using CRISPR/Cas9 strategy. Targets for Cas9 were selected using “CRISPR optimal target finder”–program from O'Connor-Giles Lab. Vector for generating dsDNA donors for homology-directed repair contains the visible marker 3xP3-DsRed. pHD-DsRed was a gift from Kate O'Connor-Giles (Addgene plasmid # 51434). dsRed gene was using for selection of flies with iab-4 deletion. (TIF) [file pgen.1007702.s003.tif]
